# Effect of 12-Month Resistance Training on Changes in Abdominal Adipose Tissue and Metabolic Variables in Patients with Prediabetes: A Randomized Controlled Trial

**DOI:** 10.1155/2019/8469739

**Published:** 2019-10-16

**Authors:** Juan Yan, Xia Dai, Jitao Feng, Xiaodan Yuan, Jianing Li, Lihong Yang, Panpan Zuo, Zhaohui Fang, Chao Liu, Cunyi Hsue, Junya Zhu, Joshua D. Miller, Qingqing Lou

**Affiliations:** ^1^Affiliated Hospital of Integrated Traditional Chinese and Western Medicine, Nanjing University of Chinese Medicine, Nanjing, Jiangsu Province, China; ^2^Nursing College, Nanjing University of Chinese Medicine, Nanjing, Jiangsu Province, China; ^3^Department of Endocrinology, The First Affiliated Hospital of Guangxi Medical University, Nanning 530021, China; ^4^Department of Endocrinology, The First Affiliated Hospital of Anhui University of Chinese Medicine, Hefei, China; ^5^University of Massachusetts Amherst, Amherst, MA, USA; ^6^Johns Hopkins Bloomberg School of Public Health, Baltimore, MD, USA; ^7^Stony Brook University Hospital, NY, USA

## Abstract

**Objective:**

To examine the effects of resistance training relative to aerobic training on abdominal adipose tissue and metabolic variables in adults with prediabetes.

**Methods:**

105 participants with prediabetes were randomized into the resistance training group (RT, *n* = 35), aerobic training group (AT, *n* = 35), and control group (CG, *n* = 35). The participants completed supervised 12-month exercise; the control group followed the primary lifestyle without exercise intervention. The primary outcomes were visceral adipose tissue (VAT) and subcutaneous adipose tissue (SAT) measured by computed tomography (CT). Secondary outcomes were body composition, lipid profile, and metabolic variables.

**Results:**

A total of 93 participants completed the study. There were nonsignificant differences between groups before intervention. After training, VAT decreased significantly in AT and RT compared with CG (*P* = 0.001 and *P* = 0.014, respectively). Although no significant difference in SAT was found across groups, SAT decreased significantly over time within each exercise group (all *P* = 0.001). Increase in muscle mass was greater in RT than that in AT and CG (*P* = 0.031 and *P* = 0.045, respectively). Compared with CG, fasting plasma glucose (FPG) decreased significantly in RT and AT (*P* = 0.003 and *P* = 0.014, respectively). There was a significant difference in the number of prediabetes who converted to diabetes among AT and RT, as compared with the control group (*P* = 0.031 and *P* = 0.011, respectively). No significant differences were observed in lipid, waist-to-hip ratio (WHR), body mass index (BMI), fasting insulin (FI), 2-hour postprandial glucose (2hPG), glycosylated hemoglobin (HbA1c), HOMA-IR, and HOMA-*β* across groups.

**Conclusion:**

Both aerobic training and resistance training are effective in reducing abdominal adipose tissue and fasting plasma glucose in adults with prediabetes. Importantly, resistance training but not aerobic training is effective in augmenting muscle mass.

**Trial Registration:**

The trial is registered with NCT02561377 (date of registration: 24/09/2015).

## 1. Introduction

Individuals with prediabetes have approximately a 30% chance of developing type 2 diabetes mellitus (T2DM) over a 10-year period [1–3]. Impaired fasting glucose (IFG), impaired glucose tolerance (IGT), and the combination of IFG plus IGT characterized by different levels of insulin resistance are prediabetes states [4]. These represent intermediate states of abnormal glucose regulation between normal glucose homeostasis and diabetes mellitus. Individuals with IFG or IGT add to the risk of evolving type 2 diabetes mellitus. However, it is not inevitable but a reversible process. Rising levels of obesity, insufficient physical activity, and sedentary behavior are independent risk factors for insulin resistance/diabetes [5]. Exercise is a recognized strategy that is vital to prevention, care, and management of type 2 diabetes and prediabetes [6, 7].

Abdominal obesity has been associated with insulin resistance, metabolic syndrome, type 2 diabetes mellitus, and cardiovascular diseases [8]. In particular, a growing body of evidence showed that increased VAT was strongly associated with insulin resistance, atherogenic dyslipidemia, and cardiovascular disease [8–10]. Regular exercise is known to reduce abdominal adipose tissue and attenuate the risk of type 2 diabetes. One study has examined that RT effectively reduced abdominal fat, especially VAT [11]. One review revealed that AT was effective in lowering VAT [12]. However, few studies on prediabetes investigated abdominal adipose tissue involving RT compared with AT. Therefore, this study is aimed at determining the effects of resistance training compared to aerobic training on abdominal adipose tissue and metabolic variables in individuals with prediabetes. We hypothesized that resistance training was the effective exercise to decrease abdominal adipose tissue.

## 2. Methods

### 2.1. Study Design and Participants

Participants were recruited primarily through four communities in Nanjing City and a hospital of Danyang from May 2014 to April 2015. One hundred and sixty-eight participants who had a history of prediabetes were recruited. All participants underwent a medical history and received a 2-hour oral glucose tolerance test (OGTT). Of them, 124 fulfilled the inclusion criteria as defined by the American Diabetes Association (ADA) [13]. Persons were eligible for inclusion as follows: (1) participants with prediabetes [13]; (2) male and no pregnant and lactating women; and (3) muscle strength ≥ IV. Persons were excluded if they had a history of diabetes, severe cardiopulmonary disease, and cognitive impairments. Furthermore, participants had to be free of other disorders that could affect their ability to complete the training. We also excluded those who had difficulties in following up and refused to participate. The final cohort included 105 participants who were willing to participate in the project. At the end of the study, all participants underwent a second OGTT to evaluate their disease conversion. This study has a double-center, randomized controlled trial (RCT) design. Participants and trainers could not be blinded to group assignment after randomization, but the study outcomes were measured by blinded assessors. The protocol (NCT02561377, registered at http://www.clinicaltrial.gov) was approved by the Ethical Committee of Jiangsu Province Hospital on Integration of Chinese and Western Medicine, and all participants were verbally introduced about the study and signed informed consent documents.

### 2.2. Measurements

#### 2.2.1. Abdominal Adipose Tissue

The method of measuring abdominal adipose tissue was reported elsewhere [14]. SAT was determined by subtracting the VAT from the total abdominal tissue. Baseline and follow-up measurements were obtained with the same instrument for all participants.

#### 2.2.2. Body Composition

Height, body weight, and muscle mass were determined using a body composition analyzer (SK-X80, Yuwell), and all participants wore light clothing and stood barefoot. BMI was calculated from weight and height (kg/m^2^). Waist circumference was measured at the midpoint between the lowest rib and the iliac crest. Hip circumference was measured with a tape measure horizontally at the place of symphysis pubis, and the posterior gluteus maximus was measured when the subject stood upright [14]. WHR was also calculated. All indicators were measured by the same trained person.

#### 2.2.3. Metabolic Variables

For biochemical testing, venous blood was collected following a minimum ten-hour fast. FI, FPG, 2hPG, HbA1c, and blood lipid levels were analyzed with a full-automatic chemiluminescence instrument (Cobas 6000, Roche). HOMA2-IR and HOMA2-*β* were calculated by a software implementation of the HOMA2 Calculator v2.2.3.

#### 2.2.4. Screening Maximum Heart Rate

Participants underwent a modified Bruce protocol treadmill test (ERS.2, Ergoline, Germany) for safety purposes before starting the exercise program, which was used to determine the maximum heart rate. We monitored the participant heart rate and blood pressure according to standard procedures, and the test was terminated when participants reached volitional fatigue throughout the treadmill test. A heart rate watch was used to monitor the heart rate of participants (Polar® A370, Polar, Finland), and each individual's HRmax was defined when they rated their dyspnea and fatigue. All participants were performed continuous monitoring of electrocardiogram during these maximal exercise tests. Participants who are found to have typical angina pectoris, syncope, systolic blood pressure drop ≥ 10 mmHg, sustained ventricular tachycardia, and ST elevation of ≥1.0 mm were excluded from the exercise program.

### 2.3. Intervention

In this 12-month research, 105 eligible participants were randomized to a resistance training group, an aerobic training group, and a control group using a table of random numbers. The control group did not exercise during the 12-month study period, following the primary lifestyle. Exercise groups were supervised by the trainers who had received professional training.

The control group was instructed to maintain their usual habits and received no structured exercise intervention.

Participants were asked to participate in a resistance training 3 days/week with a bungee cord (Huoshuai, China). There were 13 exercise pieces in the protocol: leg presses, leg extensions, chest presses, pull downs, rowing motions, calf raise, seated leg curl, shoulder presses, straight-arm forwards, straight-arm backwards, leg rotation left, leg rotation right, and abdominal crunch movements. The resistance training sessions took approximately 50 minutes to complete. The resistance for each bungee cord during the first 1-2 weeks was set by the trainers at 50% of 1RM, with frequency of 1-2/week, and 6-8 repetitions of 13 different activities and then gradually increased to 3/week, 10-15 repetitions at 60% of 1RM until completing the intervention.

Strength was assessed at the baseline by performing a one repetition maximum (1RM) test (Nitro Plus, Nautilus Inc., Vancouver, WA, USA) on the leg press and leg extension, and we accurately corrected the resistance by measuring 1RM throughout the RT intervention. The 1RM was performed between 08:30 and 10:00 a.m. After a warm-up leg press and leg extension for 5-10 repetitions on the machine were done, participants started to perform leg press and extension at a workload of 10-20% body weight. If they succeed within three attempts, the resistance weight gradually increased to the next higher level that the machine has; if one exercise was judged as “very hard” on a relative perceived exertion scale with an inability to perform it, participants would rest for 1-2 minutes, and they try again. If this level of perceived exertion was not reached, the highest loads of three attempts per exercise were reported. Maximum strength was determined for 1RM.

The aerobic training program required participants to exercise 3 days/week for 60 minutes/session (including 5-10 minutes of warm-up and 5-10 minutes of flexibility exercises). Participants were educated on aerobic exercises (aerobic dancing) with music. Participants exercised at 60 to 70% of their HRmax as determined by their treadmill test results. The heart rate was monitored during training, and the target heart rate range was progressively increased as described.

### 2.4. Dietary Regimen

All participants were asked to follow a healthy diet (55-60% carbohydrate, 15-20% protein, and 25-30% fat) throughout the study. Individualized meal plans of participants were made by a dietitian, and they were asked to record 24-hour food intake during the enrollment period, 6-month and 12-month. Dietary intakes were computed by a software of KangfuAna 4.1 plus. There was no difference in total calorie intake and macronutrient percentages across groups.

### 2.5. Statistical Analysis

Sample size calculation was performed (PASS 15, NCSS statistical software, UT). We have conducted an analysis to determine the sample size needed to detect a mean difference of 27.9 cm^2^ and an SD of 36.43 cm^2^ in VAT between the RT and AT groups [15]. The required sample size for 80% power using a 2-tailed test at alpha = 0.05 is 28 each group. Assuming a dropout rate of up to 20%, we planned to recruit 100 patients. Among 124 eligible participants, 105 finally participated in the study. Statistical analysis was performed using SPSS 22.0 software. Analysis used the intention-to-treat principle and included all randomly allocated participants. Data are presented as the mean ± standard deviation (SD), except for nonnormally distributed variables for which the group median is shown. Baseline characteristics among three groups were expressed with one-way analysis of variance (ANOVA) or a nonparametric test and a Chi quadrate test for categorical variables. Pre-to-posttraining changes were analyzed independently for comparison between each group by the Kruskal-Wallis Wilcoxon test. Repeated measures ANOVA was used to compare changes over the intervention period, with the variables assessed in the study as the dependent variable and effects for time, study group, and time-by-group interaction. The differences within-group were evaluated using a paired *t*-test or paired Wilcoxon test. Significance for all statistical tests was set at *P* < 0.05.

## 3. Results

### 3.1. Adherence

**Figure 1:**
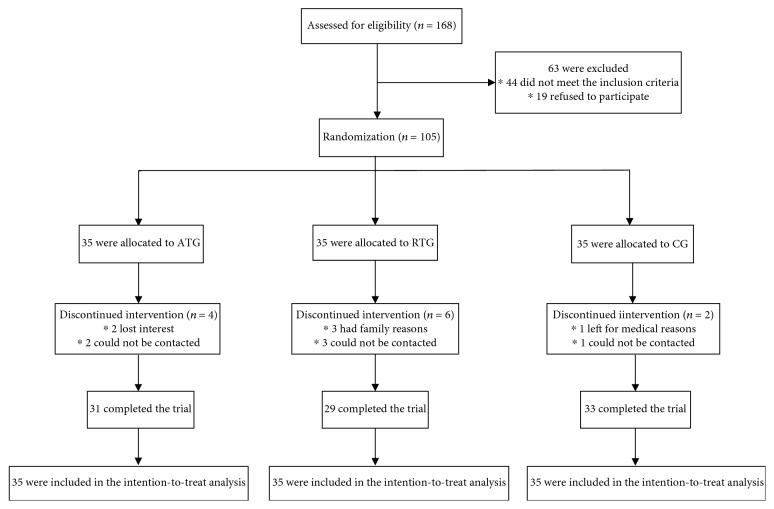
Participant flow diagram.

A total of 105 eligible participants (65 women and 40 men) were included in this study, with a mean age of 62.20 ± 7.32 years.  Figure 1 showed the flow of participants from recruitment and follow-up. A total of 93 participants completed the 12-month intervention (89% completion rate). The reasons for dropping out were family and medical conditions unrelated to the study outcomes (*n* = 3 and *n* = 1, respectively), and two participants discontinued the intervention because of losing interest. Furthermore, six participants could not be contacted. Nevertheless, all participants were included in the intention-to-treat analyses.

### 3.2. Baseline Characteristics

Baseline characteristics of the three groups were shown in Table 1. There were no differences in baseline variables across groups. Throughout the study period, no exercise-related adverse events were found.

### 3.3. Change in Body Composition

**Figure 2:**
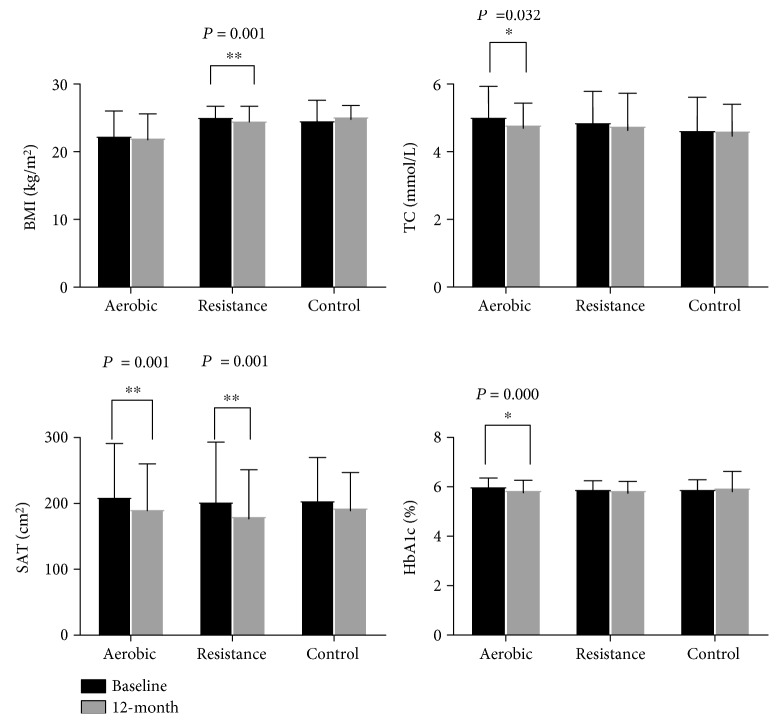
Comparisons of BMI, TC, SAT, and HbA1c at the baseline and follow-up within groups.

There were no significant changes in WHR, BMI, and SAT between groups after 12-month training. A significant increase in muscle mass (Δ0.17 ± 0.93 kg) was observed in RT compared with AT and CG (*P* = 0.031 and *P* = 0.045, respectively) (Table 2, Supplementary [Supplementary-material supplementary-material-1]). VAT decreased more in AT and RT than in CG (group-by-time interaction *P* = 0.006). Although no substantial changes were noted across groups in SAT, there was a significant difference in the change of SAT in exercise groups in within-group analyses (*P* = 0.001). BMI decreased significantly only in RT during the training period (*P* = 0.001) (Figure 2).

### 3.4. Change in Blood Lipid

There were no significant differences in total cholesterol (TC), triglycerides (TG), high-density lipoprotein (HDL), and low-density lipoprotein (LDL) between CG and each of the two exercise groups, but AT caused a 0.22 ± 0.67 mmol/L reduction in TC compared to the baseline (*P* = 0.032) (Figure 2).

### 3.5. Change in Metabolic Variables

After intervention, the reduction in FPG was found in exercise groups but not in CG (group-by-time interaction *P* = 0.004). No significant differences were noted in the following parameters: FI, 2hPG, HbA1c, HOMA2-IR, and HOMA2-*β*. In within-group analyses, decreases in HbA1c were only observed in AT (group-by-time interaction *P* = 0.033).

### 3.6. Change in Disease Conversion

After intervention, there was a significant difference in the number of prediabetes who converted to diabetes in AT and RT compared with the control group (*P* = 0.031 and *P* = 0.011, respectively) (Table 2).

## 4. Discussion

To the best of our knowledge, this study is the first 3-arm randomized controlled trial involving participants with prediabetes to investigate the effects of 12-month RT and AT compared with CG on abdominal adipose tissue and metabolic variables. Our primary findings were that exercise groups both decreased VAT (assessed by CT imaging) compared to CG, with no statistical difference between exercise groups. This finding is consistent with previous studies which demonstrated that both resistance training and aerobic training performed 180 min per week resulted in a significant reduction in VAT after 3 months [16] and 4 months [15], respectively. In contrast, Lee et al. [17] reported that significant reductions in VAT in obese adolescent girls were observed in AT but not in RT. Two reasons might account for the difference between the studies. First, it may be related to the study populations. Participants in our study were male and female adults in middle age and older prediabetes patients with average age of 62.20 ± 7.32 and BMI of 24.35 ± 3.43. The difference in gender, age, and BMI could make a big difference. Second, their study used magnetic resonance imaging (MRI) for quantifying the visceral fat volume.

Our study observed an increase in total muscle mass (0.17 ± 0.93 kg (0.4% increase)) following a 12-month exercise program using a bungee cord to perform resistance exercise at 50 to 60% of the individual 1RM. The result was consistent with previous studies [16, 18, 19], which have indicated that resistance exercise was effective in augmenting muscle mass. However, it should be noted that the increase in muscle mass is relatively minor in RT in our study. Two reasons might account for the difference compared with the previous study. First, it may be related to the equipment. We used a bungee cord to perform resistance training, while the previous study used a lift machine. Second, the difference in age could make a difference. Participants in the previous research were adolescent boys (12-18 years), but participants in our study were elderly prediabetes patients with a mean age of 62.20 ± 7.32 years. Even though it was all at 50-60% of 1RM, the absolute resistance weight in elder prediabetes patients was much lower than adolescent boys. Skeletal muscle mass is an independent predictor of prognosis in patients with chronic heart failure [20]. It was reported that lower muscle mass was associated with cardiovascular diseases [21] and resistance exercise was superior to aerobic exercise in increasing muscle mass and endurance and then improving peripheral artery wall structure and function [20]. Therefore, resistance training may have additional cardiovascular benefits than aerobic training.

In this study, we also found that both RT and AT significantly decreased FPG compared with CG. Consistently, Bacchi et al. [15] showed that FPG was similarly reduced in both exercise groups after 4 months of intervention. Ibanez et al. [22] discovered that FPG significantly decreased in adults with type 2 diabetes after 16 weeks of progressive resistance training.

After 12-month intervention, we observed that the incidence of diabetes was 8.6%, 5.7%, and 28.6% for one year in the AT, RT, and control groups, respectively. A significant difference in the number of prediabetes converted to diabetes was found in both aerobic and resistance training groups compared with the control group. This finding was consistent with the observation that the lifestyle intervention effectively reduced the incidence of diabetes [3, 23, 24]. However, it was worth mentioning that the yearly diabetes conversion rate in the control group was higher in our study (28.6%) than those in the Da Qing (12.5%) and DPP studies (11%). Possible reasons were that the average age of the patients in the present study was 60.31 ± 7.56 years, much higher than that of Da Qing and DPP studies, and all women (*n* = 65) in our study were menopause. Both age and menopause could make a big difference.

Strengths of our study included the randomized, controlled trial design and a high rate of adherence to the trial intervention (89%). Furthermore, this was a tightly controlled efficacy study, and all exercise was completed under supervision. Above all, data analysis used the intention-to-treat principle. However, the inadequate sample size may limit the power of some conclusions.

In conclusion, the findings of the present study suggest that in adults with prediabetes, both resistance and aerobic training lead to reductions in abdominal adipose tissue and fasting plasma glucose. However, only resistance training but not aerobic training is effective in increasing muscle mass.

## Figures and Tables

**Table 1:** Participant demographic and clinical characteristics at the baseline.

Characteristic		AT (*n* = 35)	RT (*n* = 35)	Control (*n* = 35)	*P* value
Age (years)		64.23 ± 5.75	62.06 ± 8.11	60.31 ± 7.56	0.08
Sex (female/male), *n*/*n*		25/10	20/15	20/15	0.36
BMI (kg/m^2^) (median 25%, 75%)		22.37 (21.37, 26.02)	25.14 (22.67, 26.71)	24.63 (21.65, 27.61)	0.34
WHR (median 25%, 75%)		0.88 (0.84, 0.92)	0.89 (0.82, 0.94)	0.89 (0.86, 0.91)	0.70
Muscle mass (kg) (median 25%, 75%)		38.90 (34.80, 42.40)	41.30 (37.90, 49.50)	40.50 (33.60, 49.00)	0.28
TC (mmol/L)		5.03 ± 0.90	4.87 ± 0.91	4.64 ± 0.97	0.22
TG (mmol/L) (median 25%, 75%)		1.23 (0.99, 2.06)	1.37 (1.00, 1.86)	0.95 (0.73, 1.57)	0.16
HDL (mmol/L)		1.43 ± 0.46	1.29 ± 0.39	1.38 ± 0.34	0.37
LDL (mmol/L) (median 25%, 75%)		2.94 (2.74, 3.42)	2.80 (2.36, 3.25)	2.61 (2.22, 3.26)	0.16
VAT (cm^2^)		150.24 ± 58.61	148.01 ± 62.36	142.18 ± 53.71	0.83
SAT (cm^2^) (median 25%, 75%)		215.88 (147.01, 246.20)	180.57 (147.42, 231.98)	205.38 (169.67, 230.63)	0.70
FPG (mmol/L)		5.83 ± 0.64	5.83 ± 0.57	5.71 ± 0.60	0.64
2hPG (mmol/L) (median 25%, 75%)		7.79 (6.82, 9.26)	7.87 (6.50, 9.86)	8.45 (7.03, 10.38)	0.56
HbA1c (%)		6.02 ± 0.34	5.91 ± 0.34	5.91 ± 0.38	0.48
HbA1c (mmol/mol) (median 25%, 75%)		42 (39, 45)	40 (39, 45)	41 (39, 44)	0.48
FI (*μ*U/mL) (median 25%, 75%)		7.89 (5.73, 11.08)	7.31 (6.30, 14.38)	8.34 (5.75, 11.33)	0.86
HOMA2-IR (median 25%, 75%)		1.02 (0.79, 1.54)	0.98 (0.87, 1.93)	1.09 (0.77, 1.46)	0.87
HOMA2-*β* (median 25%, 75%)		70.10 (53.00, 97.00)	73.70 (57.50, 106.30)	81.40 (58.30, 100.20)	0.66
Total calorie intake (kcal)		1809.61 ± 328.47	1789.34 ± 330.49	1906.04 ± 385.40	0.475
Menopause, *n*		25	20	20	0.36
Diagnostic categories, *n* (%)	IFG	17 (48.6)	10 (28.6)	11 (31.4)	0.170
	IGT	9 (25.7)	15 (42.9)	18 (51.4)	0.082
	IFG+IGT	9 (25.7)	10 (28.6)	6 (17.1)	0.505

Data are presented as the mean ± SD or median (if data are not normally distributed). AT: aerobic training; RT: resistance training; BMI: body mass index; WHR: waist-to-hip ratio; TC: total cholesterol; TG: triglycerides; HDL: high-density lipoprotein; LDL: low-density lipoprotein; VAT: visceral adipose tissue; SAT: subcutaneous adipose tissue; FPG: fasting plasma glucose; 2hPG: 2-hour postprandial glucose; FI: fasting insulin; IFG: impaired fasting glucose; IGT: impaired glucose tolerance. HOMA2-IR and HOMA2-*β* were calculated by a software of the HOMA2 model to assess insulin resistance and beta function.

**Table 2:** Before and after training between-group comparison of changes on body composition, blood lipid, and metabolic variables.

		AT (*n* = 35)	RT (*n* = 35)	Control (*n* = 35)	*P* value	*P* value	*P* value		
					Group-time interaction	Group	AT vs. RT	AT vs. control	RT vs. control
BMI (kg/m^2^)	After	22.08 (21.10, 25.58)	24.60 (21.67, 26.71)	25.22 (21.43, 26.84)	0.378	0.559	0.112	0.541	0.444
	Change	‐0.33 ± 1.04	‐0.66 ± 1.35	‐0.29 ± 1.19					
WHR	After	0.89 (0.83, 0.91)	0.87 (0.84, 0.93)	0.88 (0.85, 0.93)	0.845	0.648	0.553	0.75	0.32
	Change	‐0.006 ± 0.05	‐0.001 ± 0.04	‐0.007 ± 0.05					
Muscle mass (kg)	After	38.50 (34.80, 42.40)	41.30 (38.40, 49.50)	39.80 (34.00, 49.00)	0.112	0.230	0.031	0.892	0.045
	Change	‐0.28 ± 0.70	0.17 ± 0.93	‐0.35 ± 1.56					
TC (mmol/L)	After	4.80 ± 0.64	4.77 ± 0.96	4.62 ± 0.78	0.541	0.301	0.892	0.347	0.341
	Change	‐0.22 ± 0.67	‐0.11 ± 1.02	0.02 ± 0.55					
TG (mmol/L)	After	1.44 (1.04, 1.74)	1.46 (0.86, 1.95)	1.18 (0.97, 1.90)	0.457	0.970	0.92	0.29	0.28
	Change	0.03 ± 0.55	0.09 ± 0.51	0.19 ± 0.59					
HDL (mmol/L)	After	1.37 ± 0.66	1.31 ± 0.37	1.30 ± 0.28	0.250	0.532	0.212	0.737	0.115
	Change	‐0.06 ± 0.32	0.02 ± 0.20	0.08 ± 0.22					
LDL (mmol/L)	After	3.00 (2.72, 3.48)	2.91 (2.56, 3.49)	2.99 (2.38, 3.53)	0.352	0.235	0.934	0.171	0.176
	Change	0.07 ± 0.55	0.18 ± 0.84	0.31 ± 0.65					
VAT (cm^2^)	After	130.38 ± 49.07	135.27 ± 60.63	147.58 ± 54.77	0.006	—	0.469	0.001	0.014
	Change	‐19.87 ± 26.14	‐12.75 ± 36.88	5.40 ± 36.24					
SAT (cm^2^)	After	185.23 (147.01, 215.12)	177.33 (136.23, 200.17)	189 (166.32, 226.88)	0.665	0.839	0.545	0.087	0.056
	Change	‐18.44 ± 47.48	‐22.10 ± 43.22	‐11.02 ± 63.54					
FPG (mmol/L)	After	5.64 ± 0.66	5.66 ± 0.81	5.99 ± 0.92	0.004	—	0.473	0.014	0.003
	Change	‐0.19 ± 0.48	‐0.17 ± 0.60	0.28 ± 0.82					
2hPG (mmol/L)	After	7.00 (6.19, 8.80)	7.87 (6.10, 9.60)	8.00 (6.85, 10.58)	0.610	0.269	0.141	0.252	0.573
	Change	‐0.25 ± 1.99	‐0.16 ± 1.35	0.27 ± 3.16					
HbA1c (%)	After	5.87 ± 0.40	5.86 ± 0.36	5.97 ± 0.66	0.033	—	0.107	0.050	0.653
	Change	‐0.15 ± 0.25	‐0.06 ± 0.25	0.07 ± 0.47					
HbA1c (mmol/mol)	After	40 (38, 45)	40 (38, 43)	41 (37, 45)	0.034	—	0.106	0.066	0.727
	Change	‐1.51 ± 2.77	‐0.57 ± 2.73	0.83 ± 5.18					
FI (*μ*U/mL)	After	8.92 (5.73, 12.59)	7.89 (6.02, 11.46)	8.89 (5.65, 13.31)	0.932	0.751	0.168	0.865	0.239
	Change	0.86 ± 4.38	0.57 ± 4.54	0.90 ± 2.83					
HOMA2-IR	After	1.19 (0.75, 1.64)	1.04 (0.80, 1.47)	1.24 (0.76, 1.79)	0.892	0.772	0.23	0.902	0.182
	Change	0.11 ± 0.57	0.07 ± 0.61	0.13 ± 0.38					
HOMA2-*β*	After	81.00 (53.60, 95.50)	77.70 (56.30, 112.30)	79.00 (50.40, 110.40)	0.545	0.706	0.298	0.137	0.622
	Change	5.91 ± 26.70	5.63 ± 26.56	0.23 ± 18.56					
Total calorie intake (kcal)	After	1952.53 ± 353.47	1883.59 ± 293.88	1949.32 ± 295.72	0.315	0.621	0.697	0.211	0.387
	Change	142.92 ± 271.37	94.25 ± 217.88	43.29 ± 188.39					
Diagnostic categories after training, *n* (%)	IFG	11 (31.4)	8 (22.9)	6 (17.1)	—	—	0.420	0.163	0.550
	IGT	5 (14.3)	16 (45.7)	11 (31.4)	—	—	0.004	0.088	0.220
	IFG+IGT	6 (17.1)	1 (2.9)	5 (14.3)	—	—	0.046	0.743	0.088
	NGT	10 (28.6)	8 (22.9)	3 (8.6)	—	—	0.584	0.031	0.101
	Diabetes	3 (8.6)	2 (5.7)	10 (28.6)	—	—	1.000	0.031	0.011

The data are shown as the mean ± SD or median (if data are not normally distributed). Change: from baseline to posttraining within-group; NGT: normal glucose tolerance.

## Data Availability

The data used to support the findings of this study are available from the corresponding author upon request.

## Supplementary Materials

Supplementary Materials Supplementary Figure 1: changes in muscle mass, VAT, FPG, and 2hPBG in three groups after 12-month intervention.
