# Supplementary material for: Effect of 12-Month Resistance Training on Changes in Abdominal Adipose Tissue and Metabolic Variables in Patients with Prediabetes: A Randomized Controlled Trial
Source: J Diabetes Res. 2019 Oct 16;2019:8469739. doi: 10.1155/2019/8469739 (PMC6815994; doi:10.1155/2019/8469739)
Supplement: Supplementary Materials — Supplementary Figure 1: changes in muscle mass, VAT, FPG, and 2hPBG in three groups after 12-month intervention. [file 8469739.f1.pdf]

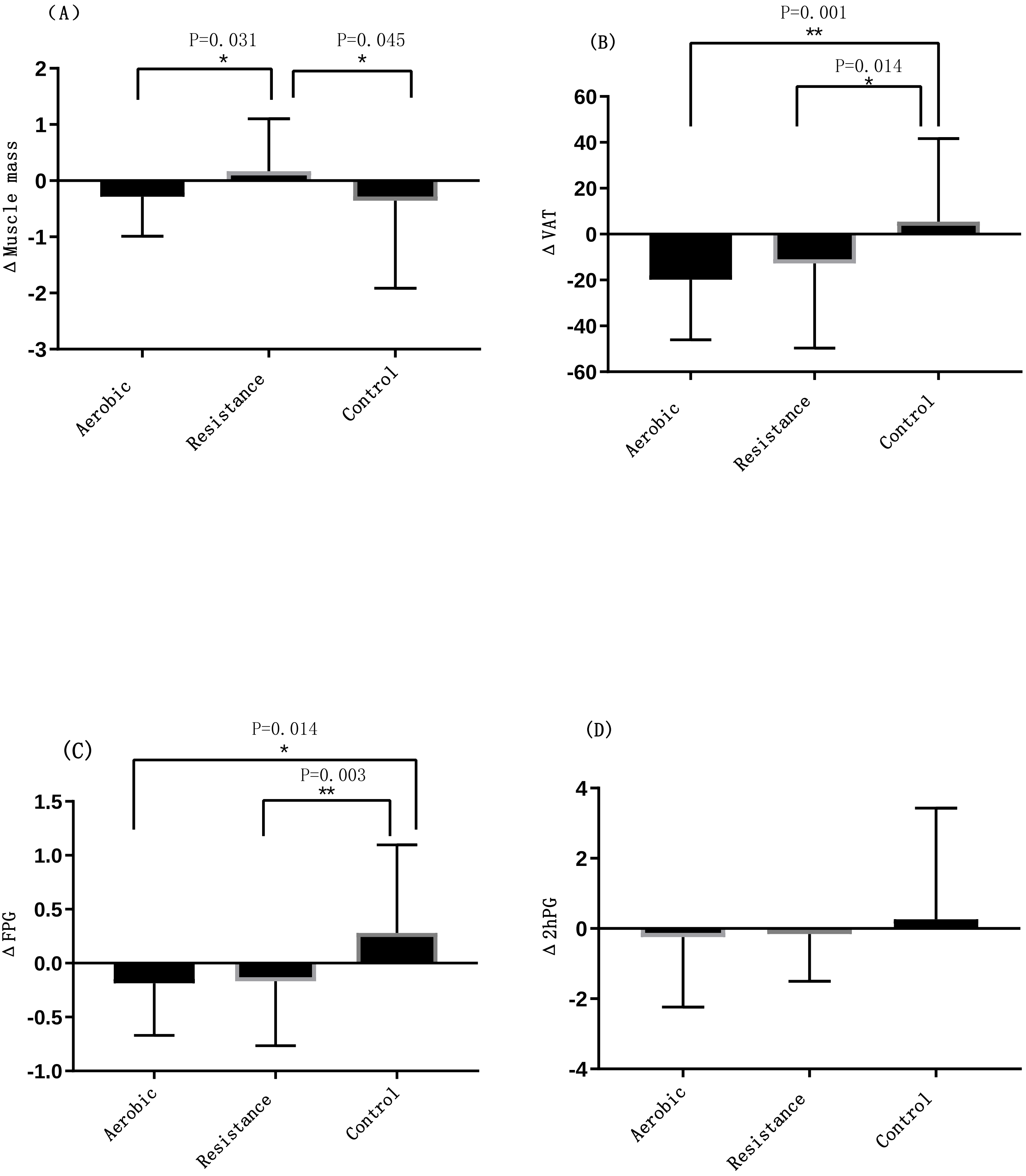

Supplementary Figure. 1 Changes in muscle mass, VAT, FPG, 2hPBG in three groups after 12-month intervention.
